# Deoxyshikonin Mediates Heme Oxygenase-1 Induction and Apoptotic Response via p38 Signaling in Tongue Cancer Cell Lines

**DOI:** 10.3390/ijms23137115

**Published:** 2022-06-26

**Authors:** Chun-Yi Chuang, Chiao-Wen Lin, Chun-Wen Su, Yi-Tzu Chen, Wei-En Yang, Shun-Fa Yang, Shih-Chi Su

**Affiliations:** 1 Department of Otolaryngology, Chung Shan Medical University Hospital, Taichung 402, Taiwan; cyi4602@gmail.com; 2 School of Medicine, Chung Shan Medical University, Taichung 402, Taiwan; 3 Institute of Oral Sciences, Chung Shan Medical University, Taichung 402, Taiwan; cwlin@csmu.edu.tw (C.-W.L.); chenyitzu0831@gmail.com (Y.-T.C.); 4 Department of Dentistry, Chung Shan Medical University Hospital, Taichung 402, Taiwan; 5 Institute of Medicine, Chung Shan Medical University, Taichung 402, Taiwan; jeff11041986@gmail.com (C.-W.S.); weienyang@gmail.com (W.-E.Y.); 6 Department of Medical Research, Chung Shan Medical University Hospital, Taichung 402, Taiwan; 7 School of Dentistry, Chung Shan Medical University, Taichung 402, Taiwan; 8 Whole-Genome Research Core Laboratory of Human Diseases, Chang Gung Memorial Hospital, Keelung 204, Taiwan; 9 Department of Dermatology, Drug Hypersensitivity Clinical and Research Center, Chang Gung Memorial Hospital, Linkou 333, Taiwan

**Keywords:** deoxyshikonin, OSCC, apoptosis, HO-1, p38

## Abstract

Deoxyshikonin (DSK), a phytochemical constituent, has been documented to elicit various oncostatic properties alone or in combination with established therapeutics. However, its role in restraining oral squamous cell carcinoma (OSCC) is mostly unclear. Here, we examined the tumor-suppressive effect of DSK and explored the molecular mechanisms underlying DSK’s activities on controlling oral cancer. Our results showed that DSK dose-dependently lessened the cell viability of tongue cancer cell lines, involving induction of cell cycle arrest at the sub-G1 phase and apoptotic cell death. Moreover, a unique signature of apoptosis-related proteins, including augmented nuclear factor erythroid 2-related factor 2 (Nrf2)/heme oxygenase-1 (HO-1) expression and caspase activation, was observed in DSK-treated tongue cancer cell lines. Furthermore, DSK-mediated upregulation of HO-1 and cleavage of caspase-9 and -3 were significantly inhibited by pharmacological blockage of p38 kinase. Collectively, these data revealed that DSK halted cell cycle progression and elicited cell apoptosis in tongue cancer cell lines, reshaping a p38-dependent profile of apoptotic proteome. Our findings provided novel insights into the therapeutic implications of a natural compound on the management of OSCC.

## 1. Introduction

Oral cancer is a common malignancy globally, with a vast majority of cases being oral squamous cell carcinoma (OSCC) [1]. In addition to a combination of surgery, radiotherapy or chemotherapy as the standard therapy of choice for this disease, a monoclonal antibody that is intended to target the epidermal growth factor receptor (EGFR) has been shown to elevate the efficacy of radiotherapy in oral cancer treatment [2]. In spite of these therapeutic strategies, the survival rate of OSCC patients remained low (~50%) [3,4,5,6], mainly resulting from cancer recurrence and dissemination. Therefore, an unmet need in improving current treatment modalities urged us to discover natural compounds with low toxicity and satisfied efficacy for tumor prevention and management.

Shikonin is a natural naphthoquinol compound extracted from the roots of purple cromwell (*Lithospermum erythrorhizon*) and has been reported to exert synergistic effects on cancer prevention/treatment in combination with established therapeutics [7]. Despite the promising potential of this phytochemical to be integrated into standard cancer care, the clinical use of shikonin as an anti-cancer drug was restricted by its low bioavailability and water solubility. To address this issue, considerable interest has been directed at several shikonin derivatives with improved pharmacological characteristics, such as their target specificity, higher water solubility, or reduced toxicity toward normal tissues. One such shikonin analogue, deoxyshikonin (DSK), was shown to exhibits a variety of oncostatic properties in a myriad of tumors through employing multiple and interrelated mechanisms. An inhibitory effect of DSK on colorectal cancer progression via downregulation of the PI3K-Akt-mTOR pathway has been documented [8]. In acute myeloid leukemia, DSK was shown to counteract cancer cell viability and glycolysis, accompanied with reduced expression and activity of pyruvate kinase M2 [9]. In addition, a recent study revealed that DSK impeded the resistance of non-small-cell lung cancer cells to chemotherapy through suppressing the expression and function of ATP-binding cassette subfamily B member 1 [10]. Although these in vitro and in vivo experimental results have indicated an anti-cancer effect of this phytochemical constituent, knowledge of the potential application of DSK on OSCC still lags behind that on the other common malignant diseases. Here, we attempted to test whether DSK effectively hampers oral cancer progression and further explored the underlying mechanisms at cellular and molecular levels. Our findings provided potential avenues for the use of a natural compound with high safety and translational value in fighting OSCC.

## 2. Results

### 2.1. DSK Dose-Dependently Decreases Tongue Cancer Cell Viability

**Figure 1:**
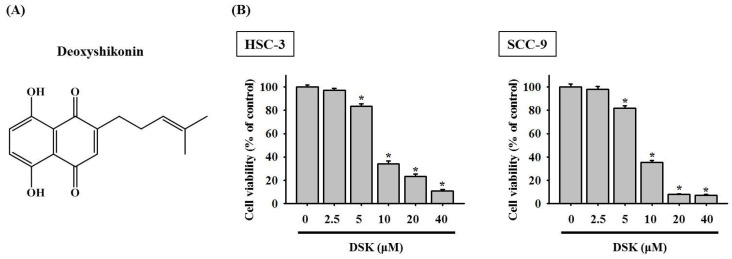
DSK reduces the tongue cancer cell viability. (**A**) Structural formula of deoxyshikonin (DSK). (**B**) The viability of tongue cancer cells in response to DSK treatment. HSC-3 and SCC-9 cells were treated with DSK at indicated concentrations for 24 h and evaluated for the cell viability. Data represent the average ± SD of three independent experiments. * *p* < 0.05, compared with untreated controls (0.1% DMSO) using Student’s *t*-test.

To test the potential cytotoxicity of DSK to tongue cancer cells, we monitored the viability of two tongue cancer cell lines, HSC-3 and SCC-9, in the treatment of DSK at various concentrations (2.5 to 40 μM). A consistent reduction in the viability of both HSC-3 and SCC-9 cells was noted in a dose-dependent manner (Figure 1). The half maximal inhibitory concentrations (IC50) of DSK were 8.995 μM in HSC-3 cells and 8.274 μM in SCC-9 cells. This finding, in accordance with the tumor suppressive features observed in other tumor types [8,9,10], points out a therapeutic potential of DSK in OSCC.

### 2.2. DSK Induces Cell Cycle Arrest and Promotes Apoptotic Responses in Tongue Cancer Cell Lines

**Figure 2:**
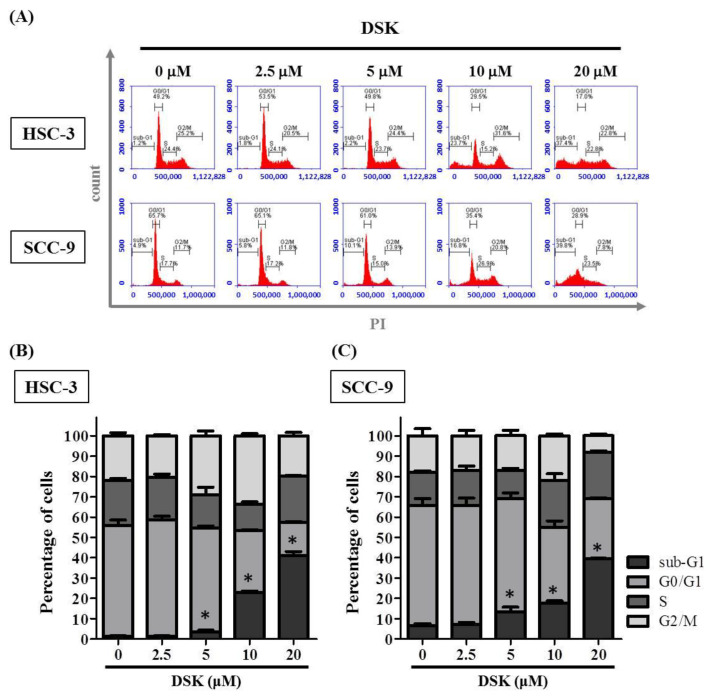
DSK induces sub-G1-phase cell cycle arrest of o tongue cancer cells. (**A**) DNA contents of tongue cancer cells (HSC-3 and SCC-9) treated with indicated concentrations of DSK for 24 h were monitored by PI staining using flow cytometry. (**B**,**C**) Quantification of cell cycle distribution in (**B**) HSC-3 and (**C**) SCC-9 cell lines. The values represent the mean ± SD of three independent experiments. * *p* < 0.05 compared with untreated controls using Student’s *t*-test.

**Figure 3:**
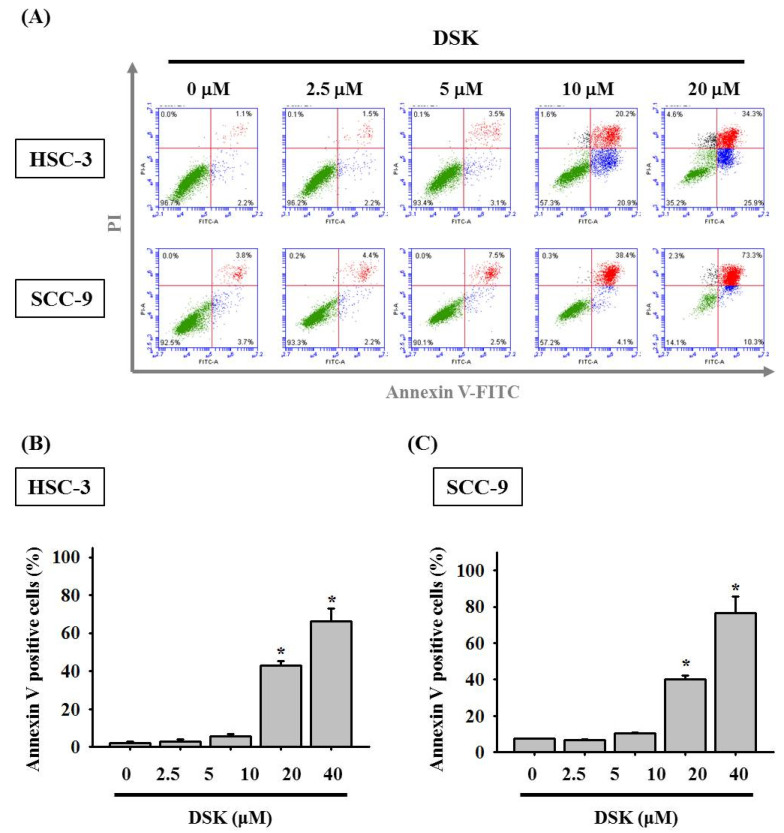
DSK promotes apoptotic responses of tongue cancer. (**A**) Double staining of PI and annexin V in tongue cancer cells after the treatment of DSK at different concentrations for 24 h was analyzed by flow cytometry. Data are representative of three separate experiments. (**B**,**C**) Quantification of apoptotic cell populations as determined by annexin-V-positive cells in HSC-3 and SCC-9 cells. Data represent the average ± SD of three independent experiments. * *p* < 0.05 compared with untreated controls using Student’s *t*-test.

Since we demonstrated that DSK counteracted tongue cancer proliferation, we further examined whether DSK exhibited its anti-cancer effect via restraining the cell cycle progression or interfering with apoptosis in tongue cancer cell lines. Through tracking DNA content, we detected a profusion of DSK-treated cells at the sub-G1 stage (Figure 2), unveiling an enhancement of sub-G1 phase cell cycle arrest by DSK. Such cell cycle arrest conceivably potentiates DSK-treated cancer cells to either trigger apoptotic pathways or undergo repair processes. Through annexin V staining, we observed that DSK augmented apoptotic responses of tongue cancer cell lines, as our flow cytometry assays exhibited an induction of both early (PI-negative/annexin-V-positive) and late apoptotic cells (PI/annexin-V-double-positive) caused by DSK treatment (Figure 3). These findings suggest that DSK halted cancer cell cycle progression and promoted cell apoptosis, implicating an oncostatic role of DSK in modulating cell biology of tongue cancer.

### 2.3. DSK Reshapes Apoptotic Proteome in Tongue Cancer

**Figure 4:**
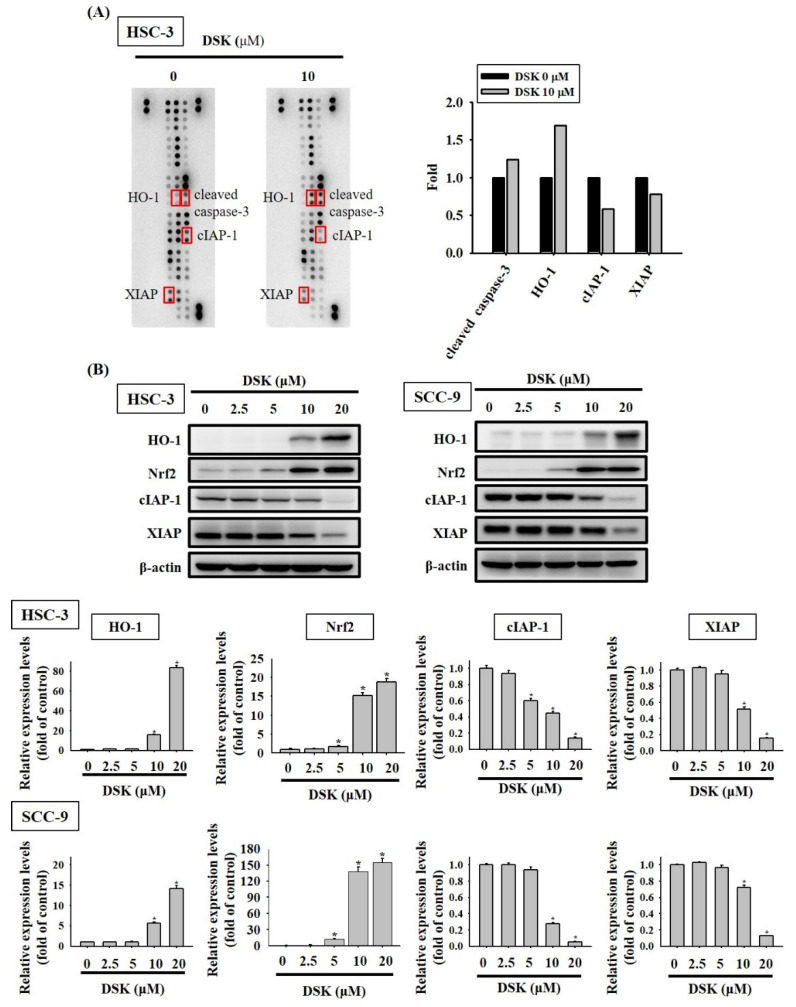
Profiling of DSK-induced apoptotic proteome in tongue cancer. (**A**) Representative images of array membranes corresponding to DSK-untreated and -treated protein expression in HSC-3 cells. Differentially expressed proteins are labeled and selected for further verification. (**B**) HSC-3 and SCC-9 cells were treated with various concentrations of DSK for 24 h and assessed for the expression of indicated apoptosis related proteins by immunoblotting. Densitometric analyses were quantified and normalized with internal controls (β-actin). The values represent the mean ± SD of three independent experiments. * *p* < 0.05 compared with untreated controls using Student’s *t*-test.

**Figure 5:**
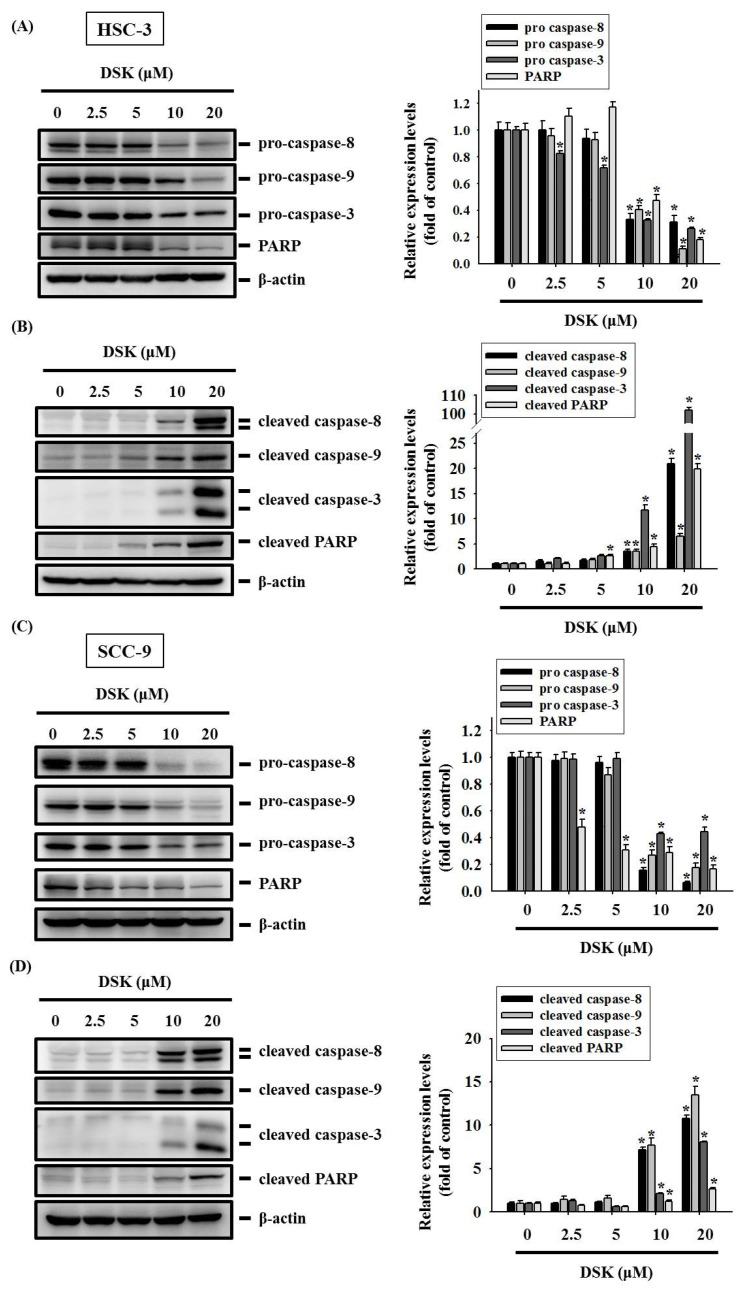
DSK stimulates the cleavage of pro-caspase-3, -8, and -9 in tongue cancer. HSC-3 and SCC-9 cells were treated with indicated concentrations of DSK for 24 h and assessed for the levels of intact (**A**,**C**) and cleaved forms (**B**,**D**) of caspases and PARP by immunoblotting with indicated antibodies. Densitometric analyses were quantified and normalized with internal controls (β-actin). Data represent the mean ± SD of three independent experiments. * *p* < 0.05 compared with untreated controls using Student’s *t*-test.

We further interrogated the molecular mechanisms by which DSK induced tongue cancer cell apoptosis through evaluating the expression of a panel of 35 apoptosis-associated proteins in DSK-treated HSC-3 cells. Our analysis of apoptotic proteome profiles showed that DSK treatment resulted in the increase in the expression of heme oxygenase-1 (HO-1) and cleaved caspase-3, whereas that caused the reduction of two apoptosis inhibitors, cellular inhibitor of apoptosis protein-1 (cIAP-1), and X-linked inhibitor of apoptosis protein (XIAP) (Figure 4A). Additional validation revealed that DSK-induced upregulation of nuclear factor erythroid 2-related factor 2 (Nrf2)/HO-1 and downregulation of cIAP-1 and XIAP were consistently noted in HSC-3 and SCC-9 cells (Figure 4B), suggesting that DSK treatment renders a specific profile of apoptotic proteome in tongue cancer. Moreover, the levels of cleaved forms of caspase-3, -8, -9, and poly (ADP-ribose) polymerase-1 (PARP) were elevated in both HSC-3 and SCC-9 cells treated with DSK (Figure 5A–D). Our data highlight a unique signature of tongue cancer apoptotic proteome induced by DSK, involving the activation of caspase cascades and exquisite orchestration of several arbitrators of cell death pathways.

### 2.4. Activation of MAPKs in DSK-Treated Tongue Cancer

**Figure 6:**
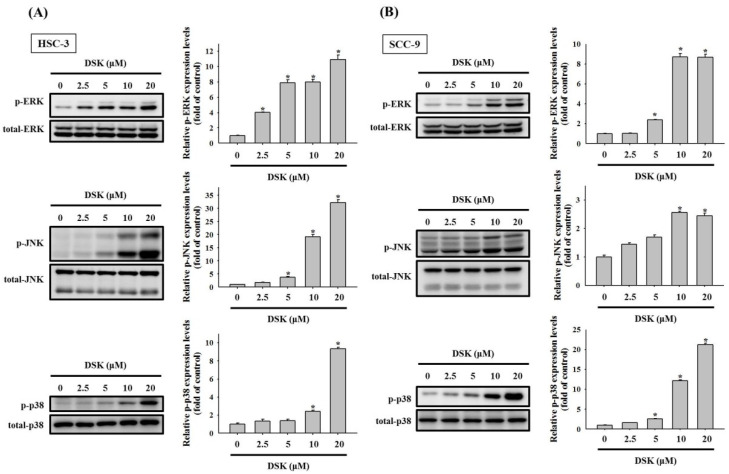
Phosphorylation of three MAPK pathway in DSK-treated HSC-3 and SCC-9 cells. HSC-3 (**A**) and SCC-9 (**B**) cells were treated with indicated concentrations of DSK for 6 h and assessed for the phosphorylation status of ERK1/2 (ERK), JNK, and p38-MAPK by immunoblotting with indicated antibodies. Densitometric analyses were quantified and normalized with total protein. The values represent the mean ± SD of three independent experiments. * *p* < 0.05 compared with untreated controls using Student’s *t*-test.

MAPKs represent a group of protein kinases that play a key role in mediating intracellular apoptotic signals in response to a great variety of external stimuli [11,12,13]. Next, we assessed the activation profiles of MAPKs in tongue cancer cells treated with DSK. Compatible findings were observed from HSC-3 and SCC-9 cells, in which exposure of DSK triggered the phosphorylation of ERK1/2, JNK, and p38 (Figure 6).

### 2.5. p38 Contributes to HO-1 Induction and Mitochondria-Mediated Caspase Activation in DSK-Treated Tongue Cancer

**Figure 7:**
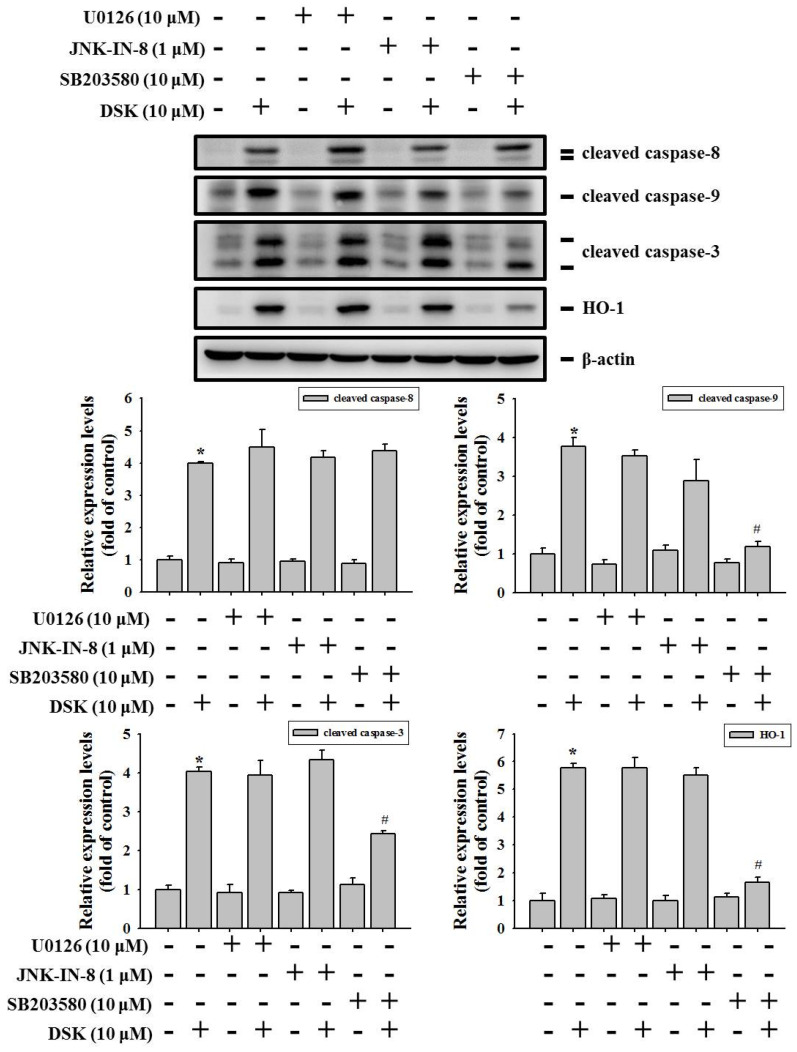
p38-MAPK mediates HO-1 induction and caspase activation in DSK-treated tongue cancer. HSC-3 cells were pretreated with indicated kinase inhibitors for 2 h and then treated with DSK for additional 24 h, followed by assessment for the levels of caspase cleavage and HO-1 expression by immunoblotting with indicated antibodies. Densitometric analyses were quantified and normalized with internal controls (β-actin). Data represent the average ± SD of three independent experiments. * *p* < 0.05 compared with untreated controls using Student’s *t*-test. # *p* < 0.05 compared with DSK-treated cells using Student’s *t*-test.

To test the potential linkage of MAPK activation with the molecular signature identified during the cell apoptosis, we further explored whether MAPKs are functionally involved in DSK-stimulated apoptotic signal pathways in tongue cancer. We demonstrated that pharmacological blockage of p38 by the treatment of SB203580 in HSC-3 cells significantly dampened DSK-induced HO-1 expression and cleavage of pro-caspase-9 and -3 (Figure 7). Yet, suppression of ERK1/2 and JNK activity by U0126 and JNK-IN-8, respectively, influenced neither the induction of HO-1 nor the activation of caspase cascades in HSC-3 cells. These results indicate a functional connection of p38-MAPK to the molecular mechanisms underlying DSK-induced tongue cancer cell death, in particular HO-1 expression level and mitochondria-mediated apoptosis.

## 3. Discussion

Although recent treatment modalities of OSCC have obtained favorable outcomes while being diagnosed at the early stage, the prognosis and overall survival of OSCC cases with the late-stage disease remain a huge challenge [14,15,16]. Thus, additional therapeutic strategies appear to be needed for the management of this disease burden. It has been commonly reckoned that natural constituents from medicinal herbs render beneficial effects on cancer treatment as combined with other established treatment regimens [17,18,19,20,21]. Here, we demonstrated that DSK, one derivative of a natural naphthoquinol compound (shikonin) extracted from the roots of *Lithospermum erythrorhizon*, showed an inhibitory effect on the cell viability of tongue cancer, involving the promotion of cell cycle arrest and apoptosis. Further exploration of molecular mechanisms behind DSK’s actions revealed that DSK-stimulated apoptosis was accompanied by p38-mediated HO-1 upregulation and caspase activation. Our results, for the first time, implicate a usefulness of this phytochemical in controlling oral carcinogenesis. 

Cumulative evidence has suggested that shikonin and its derivatives, employing various mechanisms, possess diverse anti-cancer features against numerous tumor types [22]. Modifications of shikonin can lead to the improvement of water solubility and tumor-suppressive potency. In examining a series of shikonin derivatives for their inhibitory effects on colorectal cancer progression, DSK was found to induce cell apoptosis through the attenuation of PI3K-Akt-mTOR signaling pathways [8]. By also suppressing the Akt-mTOR pathway, DSK has been shown to counteract the viability and glycolysis of acute myeloid leukemia cells [9]. Inhibition of tumor glycolytic activities is known to be particularly effective against cancer cells with mitochondrial defects or under hypoxic conditions, which are frequently associated with cellular resistance to conventional chemotherapy and radiation therapy [23]. In accordance with this finding, a recent study reported that DSK impeded the resistance of non-small-cell lung cancer cells to chemotherapy through suppressing the expression and function of ATP-binding cassette subfamily B member 1 [10]. In addition to being effective in combination with conventional cancer treatment remedies, DSK and other shikonin derivatives have been shown to bind to EGFR kinase domain and to halt EGFR phosphorylation, thereby boosting the efficacy of an EGFR antagonist, erlotinib, on glioblastoma treatment [24]. As a matter of fact, EGFR amplification is prevalent in many tumor types, including head and neck cancer [25]. To fill the gap regarding the effect of shikonin derivatives on tongue cancer, we demonstrated that DSK treatment rendered a unique apoptotic proteome profile in tongue cancer, involving induction of Nrf2/HO-1 levels and caspase activation. Even though Nrf2/HO-1 overexpression was commonly detected in malignancies and frequently associated with a cytoprotective effect on carcinogenesis [26], anti-proliferative or pro-apoptotic functions of Nrf2/HO-1 induction were also documented [27,28,29,30,31,32]. Such contradictory findings imply that Nrf2/HO-1 might function in a tissue-specific manner and act as a promotor of cell apoptosis in OSCC instead of a blocker of cancer cell death. Moreover, our results indicated that pharmacological blockade of p38 disturbed DSK-mediated apoptotic proteome, in particular the upregulation of Nrf2/HO-1 and cleavage of pro-caspase-3 and -9. Compatible results were observed from another study where p38 activation and reactive oxygen species (ROS) accumulation mediated by another shikonin derivative, acetylshikonin, were shown to contribute to apoptotic cell death in OSCC [33]. Furthermore, Hseu et al. reported that flavokawain B induces HSC-3 cells apoptosis through upregulation of the Nrf2/HO-1 pathway [34]. Our data, together with the findings from other groups, suggest that DSK interfered with cellular redox homeostasis, manifested as a p38-dependent signature of apoptotic proteome, including induction of Nrf2/HO-1 expression and mitochondria-mediated caspase cleavage (caspase-9 and -3), ultimately resulting in elevated cell death of tongue cancer. 

Our results reveal a tumor-suppressive effect of DSK on tongue cancer progression through eliciting cancer cell apoptosis. Yet, extra work is needed to manage some limitations of our study. One issue is that, even though we showed an induction of tongue cancer apoptosis by the treatment of DSK in in vitro experiments, the capacity of this phytochemical may be different after being ingested and absorbed in in vivo settings. Additional animal experiments are needed to verify the translational value of DSK on combating oral carcinogenesis. Another concern is that both HSC-3 and HCC-9 cells examined in this study were originated from tumors of the tongue. One of the challenges in OSCC research has been associated with a variety of anatomical sites that comprise this malignancy, such as buccal mucosa, tongue, lip, and gingiva. Different sites of oral cancer have distinct mutational signatures, etiology, and survival rate [35,36,37,38]. Use of tongue cancer cell lines derived from additional anatomical sites to explore DSK’s actions is warranted to strengthen the clinical relevance. 

## 4. Materials and Methods

### 4.1. Cell Culture and Reagents

Human tongue cancer cell lines, SCC-9 and HSC-3, were obtained from the American Type Culture Collection (Manassas, VA, USA) and propagated using Dulbecco’s Modified Eagle Medium/Ham’s F12 Nutrient Mixture (DMEM/F12; Life Technologies, Grand Island, NY, USA) supplemented with 10% fetal bovine serum (FBS) (Gibco, Grand Island, NY, USA), 1.2 g/L sodium bicarbonate (Sigma-Aldrich, St. Louis, MO, USA), 15 mM HEPES (Sigma-Aldrich), and 1% penicillin/streptomycin (Sigma-Aldrich) at 37 °C in a humidified atmosphere of 5% CO_2_ [39,40]. Deoxyshikonin (DSK), of HPLC grade with ≥98% purity, was obtained from ChemFaces (Wuhan, Hubei, China) and dissolved in DMSO (Sigma-Aldrich).

### 4.2. Assessment of Cell Viability

Cell viability was measured with a microculture tetrazolium (MTT) colorimetric assay (Sigma-Aldrich) as previously described [41]. Cells were treated with different concentrations of DSK in 24-well plates for 24 h. The viability of tongue cancer cells was estimated according to the generation of formazan following solubilization with isopropanol, which was measured spectrophotometrically at 563 nm in a spectrophotometer (DU640, Beckman Instruments, Fullerton, CA, USA).

### 4.3. Flow Cytometry

Levels of cellular DNA and membrane annexin V were monitored to determine the cell cycle and apoptotic response of DSK-treated tongue cancer cells, respectively, by using flow cytometry as described previously [42]. In brief, 3.5 × 10^5^/well HSC-3 and 3 × 10^5^/well SCC-9 cells in 6-well plates in response to various concentrations of DSK for 24 h were stained with PI (Invitrogen, Carlsbad, CA, USA), and the analyses of cell cycle distribution were performed with a BD AccuriTM C6 Plus personal flow cytometer (BD Biosciences, San Jose, CA, USA). For assessing cell apoptosis, annexin V in the external surface of plasma membrane was detected by using an FITC-labeled Annexin-V/PI Apoptosis Detection kit (BD Biosciences, San Jose, CA, USA) following the manufacturer’s instructions. Cells in the treatment of indicated concentrations of DSK for 24 h were harvested, subjected to staining with FITC-conjugated annexin V and PI for 20 min in the dark, and analyzed via flow cytometry.

### 4.4. Profiling of Apoptotic Proteome

Proteome of apoptotic tongue cancer cells in response to DSK treatment was analyzed by using a Proteome Profiler Human Apoptosis Array Kit (R&D Systems, Minneapolis, MN, USA), allowing to assess a panel of 35 key apoptotic proteins simultaneously. Then, 200 μg of protein lysate from DSK-treated and -untreated HSC-3 cells was applied to each array set according to manufacturer’s instructions. Quantification for the signal intensity of spots was conducted by using Image-Pro Plus software, and measured pixel density was normalized to corresponding reference array spots.

### 4.5. Immunoblotting

Tongue cancer cells were pretreated with or without an ERK inhibitor (U0126, Cell Signaling Technology, Danvers, MA, USA), a JNK1/2 inhibitor (JNK-IN-8, Calbiochem, San Diego, CA, USA), or a p38 inhibitor (SB203580, Calbiochem, San Diego, CA, USA) for 2 h and maintained in the presence or absence of DSK for 24 h. Total protein lysates (20 μg) were harvested and subjected to SDS-PAGE analyses [43,44]. Specific antibodies targeting the following molecules were used for detection: Anti-cleaved Caspase-8 (#9496), Anti-cleaved Caspase-9 (#9505), Anti-cleaved Caspase-3 (#9664), Anti-Caspase-8 (#9746), Anti-Caspase-9 (#9502), Anti-PARP (#9542), Anti-Phospho-Erk1/2 (#4370), Anti-Erk1/2 (#9102), Anti-Phospho-JNK (#4668), Anti-JNK2 (#9258), Anti-c-IAP1 (#7065), and Anti-XIAP (#2045) antibodies from Cell Signaling Technology (Danvers, MA, USA); Anti-Caspase-3 (610323), Anti-phospho-p38 (612281), and Anti-p38 (612168) antibodies from BD biosciences (San Jose, CA, USA); Anti-β-actin (ab8226) and anti-HO-1 (ab68477) antibodies from Abcam (Cambridge, UK); Anti-Nrf2 (GTX55732) antibodies from GeneTex (Irvine, CA, USA); and HRP-conjugated secondary antibodies (Dako Corporation, Carpinteria, CA, USA). Densitometry data of immunoblots were generated and analyzed by ImageJ software.

### 4.6. Statistical Analysis

For all measurements, data are shown as mean ± standard deviation (SD) from at least three separate experiments. A *p*-value of <0.05 by Student’s *t*-test was used to determine significant difference.

## 5. Conclusions

In conclusion, we showed that DSK halted cell cycle progression and promoted apoptotic responses in tongue cancer, accompanied by HO-1 induction and caspase activation through the p38-MAPK pathway. Our findings provide new insights into the therapeutic implications of a natural compound, DSK, on the management of OSCC.

## Data Availability

The data presented in this study are available on request from the corresponding author.
